# Improving the Confidence of Resident Doctors in Managing Ligature Emergencies on Inpatient Mental Health Wards

**DOI:** 10.1192/bjo.2026.11322

**Published:** 2026-06-30

**Authors:** Alice Edwards, Helena Keep

**Affiliations:** East London NHS Foundation Trust, London, United Kingdom

## Abstract

**Aims:**

Strangulation is the commonest method of suicide for both males and females, accounting for 56.6% of completed suicide in England and Wales in 2024. The inpatient mental health setting is a high-risk period for suicide attempts and completed suicide. A mental health admission should offer service users a contained environment where potential risks are well managed. However, there is no formal teaching to core psychiatry trainee doctors at East London NHS Foundation Trust regarding managing ligature emergencies on the mental health wards. Therefore the aim of this project was to improve confidence of resident doctors in managing ligature emergencies on inpatient mental health wards.

**Methods:**

A 1-hour teaching session on managing ligature emergencies was given to core trainee psychiatry doctors during their weekly teaching afternoon, which included theoretical teaching and a clinical simulation. This was run on three occasions across two sites at East London Foundation Trust. A pre- and post-session questionnaire usinga 5-point Likert scale: 1 (Not confident) to 5 (Completely confident) was formulated and provided to core traineepsychiatry doctors (n=20) to assess confidence across three domains of strangulation management: (1) removing a ligature, (2) an A to E assessment of a person who has tied a ligature and (3) recognising the red flags after a ligature. The self-perceived competence score was calculated as a percentage for each domain.

**Results:**

On average, scores improved by 2.33 (domain 1), 1.3 (domain 2) and 1.58 (domain 3). When we categorised into binary ‘Not confident’ (scores 1–3) and ‘Confident’ (scores 4–5) there was an improvement in confidence by 66.25% in domain 1, 49.6% in domain 2 and 83.75% in domain 3. The improvement between these categories was statistically significant with a p value of <0.05. Only one person had received any prior training regarding ligatures.

**Conclusion:**

This simulation resulted in doctors’ confidence improving significantly in all three domains. This pilot project demonstrates there is a gap in training for doctors and that peer-lead simulation is an effective way to address this. This project is live and we are continuing to complete more cycles of data. With each cycle, we are refining the teaching and the simulation based on the feedback. We aim to integrate the strangulation teaching session into the resident doctor’s induction across all sites, with the plan for regional rollout in the future.

